# A multidimensional procurement literacy instrument: Development and validation among undergraduate procurement students in Ghana

**DOI:** 10.1371/journal.pone.0341565

**Published:** 2026-02-03

**Authors:** Priscilla Boafowaa Oppong, Daniel Ofori, Phanuel Wunu, Edmond Yeboah Nyamah, Evelyn Yeboah Nyamah, Justice K. G. A. Boateng, Gloria K.Q. Agyapong

**Affiliations:** 1 Department of Marketing and Supply Chain Management, School of Business, University of Cape Coast, Cape Coast, Ghana; 2 Department of Education Programmes, College of Distance Education, University of Cape Coast, Cape Coast, Ghana; Polytechnic Institute of Setúbal: Instituto Politecnico de Setubal, PORTUGAL

## Abstract

Procurement remains one of the central functions in public- and private sector governance, yet relatively little attention has been given to how procurement capabilities develop during undergraduate education. This study addresses this gap by developing and validating an instrument to measure Procurement literacy of undergraduate students in Ghana. Drawing on CIPS Global Professional Standards and other policy frameworks, this study initially conceptualised six domains of procurement literacy. The initial instruments were refined through expert review before being administered to a sample of 554 students from selected public universities. The structure and psychometric properties of the administered instruments were examined using exploratory and confirmatory factor analyses. In total, a 30-item instrument grouped into five domains emerged: Ethical Procurement Practice, Procurement Planning and Decision-Making, Supplier and Contract Management, Digital and E-Procurement competency, and Legal and Policy Knowledge. The final 30-item scale demonstrated strong internal consistency, satisfactory convergent and discriminant validity, and measurement invariance across academic levels. Predictive validity was examined using the intention of students’ behavioural intention to engage in ethical procurement practices, with all dimensions showing positive and statistically significant associations. Differences in procurement literacy were also observed at academic levels, with effect sizes ranging from small to moderate. Harman’s single-factor test (48.5%) indicated that common method bias was not a threat. The findings suggest that procurement literacy can be measured reliably at the undergraduate level and that the instrument offers a practical tool for curriculum evaluation, instructional planning, and early capability assessment in procurement education.

## 1. Introduction

Procurement as a key strategic function of organisations is the overarching process of acquiring goods, works, and services. It is critical for ensuring transparency, efficiency, and value for money in public sector management. The mainstreaming of the procurement process was started by the World Bank in the late 1990s and conditioned aid to the adoption of a transparent and competitive procurement system in the early 2000s [1,2]. This has paved the way for several countries to pursue procurement reform, leading to the enactment of laws and the alignment of national systems with international frameworks such as the United Nations Commission on International Trade Law (UNCITRAL) Model Law and the World Trade Organisation (WTO) Government Procurement Agreement [3–5].

As countries strengthen regulatory procurement regimes to mitigate corruption and improve procurement processes, the need to have a competent workforce to manage the procurement functions becomes critical. Ghana, like many other countries, has pursued public procurement reforms through legislation and enacted the Public Procurement Act, 2003 (Act 663, as amended by Act 914 and Act 1139), to strengthen its procurement system. In line with the reforms, universities began to offer undergraduate programmes in procurement at the university level to train procurement professionals. Notwithstanding the progress made, procurement infraction challenges, such as non-compliance, ethical misconduct, and suboptimal decision-making, continue to be reported by the Auditor-General [3,6]. The World Bank andOrganisation for Economic Development (OECD) (attribute these infractions to deficiencies in knowledge, skills, and ethical dispositions of procurement personnel [3,7,8]. A central question revolves around how well procurement graduates are equipped with functional areas, including legal knowledge, procedural planning, transparency and accountability, supplier engagement, ethical reasoning, and digital skills. A practical way to address the readiness of graduates or students is to assess their level of literacy in these functional areas. Unfortunately, the existing literature on procurement education often focusses on ethics or legal frameworks but lacks comprehensive tools to assess students’ competencies [3,9–11]. This leaves a critical gap in understanding whether procurement education is adequately equips students for the professional demands of the field.

Contrary to the traditional notion of literacy being the ability to read and write, the contemporary view presents literacy as encompassing the capacity to apply knowledge, reason critically, and perform effectively in specialised contexts [12]. In the field of procurement, literacy involves a combination of knowledge, practical skills, and ethical awareness necessary to manage procurement processes. The constructivist learning theory [13] emphasises that meaningful learning occurs when learners connect new knowledge with prior experience, which structured education in procurement aims to facilitate. In support of this, competency-based education theory promotes multidimensional assessment as a means of capturing the skills and attributes of the real-world required in professional disciplines [14]; in addition human capital theory [15] suggests that investments in education and training improve individual productivity and contribute to national development. Developing and validating a procurement literacy instrument is therefore not only essential for professional capacity building, but also for aligning educational outcomes with national policy and institutional goals. Despite its importance, multidimensional validated tools for measuring procurement literacy of procurement students are missing in the literature.

This gap in the literature poses two challenges in the procurement education and practice. Firstly, curriculum design in procurement education mostly lacks feedback mechanisms to assess whether students acquire the necessary practical competencies before entering the workforce. Secondly, in the absence of validated tools for measuring procurement literacy, educators and policymakers are unable to effectively evaluate programme outcomes to identify skill deficits and benchmark student performance over time. The existing literature focusses predominantly on curriculum content and legal or policy reforms in procurement education, with limited attention to assessing how well students develop the necessary competencies in legal, procedural, ethical, and digital domains [11,16–19].

This study develops and validates a multidimensional instrument designed to measure procurement literacy among undergraduate students in Ghana, focussing on competencies aligned with the Chartered Institute of Procurement and Supply (CIPS) Global Standard (UK 4.0). Within the CIPS Global Standard, proficiency levels 1–4 reflect a progression from tactical support functions, through operational and managerial responsibility, to the ability to contribute to strategic procurement planning and decision-making. The instrument targets Level 1 (Tactical Support) to Level 4 (Strategic), reflecting the typical scope of undergraduate procurement education. This is based on well-established theoretical models of procurement capability development and adheres to key policy and professional standards, including the Ghana Public Procurement Act (Public Procurement Authority, 2003) [20], the OECD Recommendation on Public Procurement [21], and the CIPS Global Standard for Procurement and Supply [22]. These frameworks collectively inform the competency domains and performance expectations relevant to procurement education and practice at the undergraduate level. Informed by the framework, the instrument started with a broad conceptual mapping covering six core domains essential for procurement practice: legal and policy knowledge, procurement planning and decision-making, supplier and contract management, transparency and accountability, ethical procurement practices, and digital/e-procurement competence. These domains align with key competency areas in the CIPS framework and aim to assess the progressive development of students’ procurement capabilities throughout their academic careers. However, it is important to note that this initial conceptualisation served as a starting point rather than a fixed structure. Consistent with the process of development of psychometric scale, the final configuration of dimensions was determined through empirical testing. As reported later, one of the initially proposed domains (transparency and accountability) did not demonstrate sufficient empirical distinctiveness and was therefore excluded during the validation process, bringing the final validated instrument to five.The study also examines the ethical disposition of the students to improve the behavioural and predictive validity of the instruments.

This study, using rigorous psychometric tests such as content validation and factor analysis, aims to contribute to theory and practice. It helps in creating standardised metrics for procurement education and paves the way for research on procurement skills, ethics, and educational results in developing regions.

The rest of the study is arranged as follows: Section 2 introduces the research the conceptualisation of procurement literacy. Section 3 presents the theoretical underpinnings while section 4 introduces the procure literacy dimensions. Ethical intention was introduced in section 5 as a criterion for predictive validity in procurement literacy. Section 7 describes the methodology, the study research design, study participants, analytical techniques, and ethical considerations. Results and discussions are presented in section 8 and the conclusion in section 9.

## 2. Conceptualising procurement literacy

The concept of procurement literacy is underdeveloped in the academic literature, but based on frameworks for training procurement professionals, it can be conceptualised as a multidimensional construct encompasses knowledge, skills, and attitudes essential to effectively participate in procurement decision-making [3,7]. These include understanding legal and regulatory frameworks, strategic planning, managing supplier relationships, upholding ethical and accountability standards, and appreciating digital platforms for managing the procurement process. Learning from frameworks on functional literacy [23] and financial literacy [24] we define procurement literacy as *the knowledge, skills and ethical competencies required to understand, manage and participate in procurement systems effectively. Unlike to* professional procurement competence, procurement literacy is conceptualised as a foundational capability that reflects an individual’s understanding, judgement, and ethical orientation in relation to procurement processes. This framing positions procurement literacy as a precursor to professional competence, shaped by formal education rather than occupational experience.

From the lens of public sector procurement, compliance, transparency, and value for money are actualised through procurement literacy [25]. Within an academic setting, it offers an opportunity to evaluate curricula or measure the extent to which students have acquired competencies necessary for professional success. However, the existence of validated instruments to assess procurement literacy of students has received little attention in the literature, especially in sub-Saharan Africa, where formal procurement education is still emerging [17,26,27]; highlightingthe urgent need for robust; andmultidimensional tools to assess procurement literacy in educational contexts.

## 3. Theoretical underpinnings

We propose three theoretical frameworks for the development and validation of the procurement literacy instruments: constructivist learning theory, competency-based education and human capital theory. First, the constructivist learning theory of [13] posits that learners build knowledge actively by engaging in tasks that connect new concepts to prior experiences. It emphasises that, through guided instruction, peer interaction, and scaffolded learning experiences, students are better equipped to internalise concepts and apply them to real-world challenges ( [28,29]. In the context of procurement education, this theoretical orientation underscores the relevance of learning environments that integrate theory with authentic practice-based scenarios. We also draw on competency-based education (CBE) [30,31], which suggests that learning should be measured by the demonstration of clearly articulated competencies that reflect industry and professional standards. This model aligns with calls for procurement education for outcome-oriented curricula that promote the mastery of knowledge, ethical reasoning, strategic thinking, and digital literacy [3,7]. It also provides a methodological rationale for constructing and validating an instrument that captures multiple dimensions of procurement literacy in observable and assessable forms.

Finally, we dwell on human capital theory [15], corroborated by [32], which offers an economic rationale for procurement literacy by viewing education as a strategic investment that enhances individual productivity and institutional effectiveness. It views procurement literacy as more than academic, it is a capability that equips students with employable skills, promotes compliance and accountability in public procurement, and supports national development goals, especially in developing economies.

The three theoretical foundations justify the multidimensional conceptualisation of procurement literacy as encompassing cognitive (legal and policy knowledge), behavioural (skills, planning, and ethical dispositions), and technological (digital procurement competence) dimensions. This theoretical framwork provides the foundation for the development of a valid and reliable instrument that addresses an existing gap in the assessment of procurement education outcomes. In addition, these theoretical perspectives shaped the design of the instrument. For example, constructivist learning theory guided the development of items that assess applied understanding and contextual judgement rather than factual recall, reflecting how students engage with procurement scenarios in practice-based learning environments. The competency-based education also informed the organisation of items into domains aligned with clearly articulated capability areas and progression expectations. Finally, human capital theory further justified the inclusion of domains such as digital and e-procurement competence, recognising these skills as productive assets that enhance employability and institutional effectiveness. Together, these perspectives provided a coherent foundation for both the conceptualisation and operationalisation of procurement literacy in this study.

## 4. Dimensions of procurement literacy

The conceptualisation of procurement literacy was informed by policy instruments, educational standards, competency frameworks, and procurement capability models. These include the Chartered Institute of Procurement and Supply (CIPS) Global Standard for Procurement and Supply [22], the World Bank’s Procurement Competency Framework [33], and the OECD Recommendation on Public Procurement [21]. The dimensions of the instruments reflect competency expectations aligned with Levels 1–4 of the CIPS proficiency hierarchy. The CIPS Global Standard for Procurement and Supply emphasises on four proficiency Levels, representing a progressive development of capability. Level 1 covers basic tactical support and compliance. Level 2 progresses to operational tasks such as sourcing and routine decision-making. Level 3 becomes to a managerial focus, requiring analytical oversight of contracts and suppliers. Finally, Level 4 centres on strategic leadership, aligning procurement with organizational goals and long-term value. These levels encompass tactical and operational competencies expected of graduates from undergraduate procurement programmes, which aim to build the foundational capabilities necessary for professional practice. It also aligned with policy and legal frameworks, including the Ghana *Public Procurement Act* (Act 663, as amended by Act 914 and Act 1139). The instrument covered the core domains necessary for compliance; accountability, and strategic contribution within the procurement function and was supported by competency-based education (CBE) theory and competency modelling approaches in the professional development literature [14,34]. These domains capture distinct but interconnected aspects of procurement proficiency that must be reliably assessed through psychometric validation.

Based on a review of the current literature and competency standards, the following six core dimensions were initially identified and presented in Table 1.

**Table 1:** Dimensions of Procurement Literacy.

Dimension	Domain covered	
*Legal and Policy Knowledge*	This dimension covers the awareness and understanding of the legal frameworks, procurement thresholds, regulatory responsibilities, and institutional structures that govern public procurement. It serves as the foundational layer of procurement competence, shaping how professionals navigate procedures and maintaind compliance.	Ghana Public Procurement Act, 2003 (Act 663, as amended by Act 914 and Act 1139); CIPS Global Standard for Procurement and Supply [22]; OECD Recommendation on Public Procurement [21]; [9].
*Procurement Planning and Decision-Making*	This dimension focusses on the ability to assess procurement needs, prepare budgets, choose suitable procurement methods, and plan realistic timelines and specifications. It plays a vital role in aligning procurement decisions with an broader strategic objectives of an organisation. [7]	CIPS Global Standard for Procurement and Supply [22]; OECD Recommendation on Public Procurement 21]; Checklist for Supporting the Implementation of the OECD Recommendation of the Council on Public Procurement [7]
*Supplier and Contract Management*	This dimension covers the key competencies required to evaluate bids, select suppliers, negotiate contracts, monitor performance, manage disputes, and handle contract variations. These skills are essential to achieve value for money and building strong, sustainable relationships with suppliers.	CIPS Global Standard for Procurement and Supply [22]; World Bank Procurement Framework [33]; OECD Recommendation on Public Procurement [21]; [35]
*Transparency and Accountability*	This dimension highlights the importance of clear documentation, readiness for audits, and the use of mechanisms such as whistleblower channels. It reflects a commitment to openness and accountability throughout the procurement process [7].	OECD Recommendation on Public Procurement [21]; Ghana Public Procurement Act, 2003 (Act 663, as amended); OECD Checklist for Supporting the Implementation of OECD Recommendation of the Council on Public Procurement (2016)
*Ethical Procurement Practice*	This dimension involves the ability to recognise ethical dilemmas, manage conflicts of interest, and act consistently with integrity. It reinforces the moral responsibility of procurement professionals to uphold fairness and public trust.	CIPS Global Standard for Procurement and Supply [22]; OECD Recommendation on Public Procurement [21]; Ghana Public Procurement Act, 2003 (Act 663, as amended); [36–38]
*Digital and E-Procurement Competence*	This dimension captures the ability to use digital platforms for tendering, maintain secure procurement systems, and manage electronic records efficiently. It underscores the growing importance of digital readiness in modern procurement practice.	CIPS Global Standard for Procurement and Supply [22]; World Bank Procurement Framework [33] (2016); OECD Digital Government Policy Framework [39–41]

**Note:** These dimensions represent the *initial conceptual framework*. The final validated instrument retained five dimensions after exploratory and confirmatory factor analysis.

## 5. Ethical intention as a criterion for predictive validity in procurement literacy

Ethical intention refers to an individual’s willingness to engage in morally appropriate behaviour and is often used as a benchmark for evaluating the effectiveness of professional training and competence [42,43]. In procurement education, it serves as a meaningful criterion for assessing whether technical knowledge translates into ethical behaviour.

Each dimension of procurement literacy, including Ethical Procurement Practice, Procurement Planning and Decision Making, Supplier and Contract Management, Digital and Electronic Procurement Competency, and Legal and Policy Knowledge, has a direct or indirect influence on ethical conduct. For example, ethical practices have been shown to correlate with ethical intentions [44], while planning and legal knowledge provide a structured decision framework that helps reduce unethical discretion [45,46].

Digital competence also improves transparency, limiting opportunities for misconduct [47,48]. Supplier and contract management introduces practical ethical challenges, and competence in this area correlates with moral decision making in procurement [16],

Given these relationships, using ethical intention to assess the predictive validity of procurement literacy constructs is both theoretically justified and empirically supported. It provides insight into whether knowledge-based competencies foster ethical readiness among students and future practitioners [49,50].

## 6. Methodology

### 6.1. Research design

This study employed a quantitative cross-sectional survey design to develop and validate a multidimensional instrument to assess procurement literacy among undergraduate students in Ghana. The approach allowed for the systematic collection of data and the evaluation of psychometric properties using established statistical procedures.

### 6.2. Participants and sampling

A total of 554 undergraduate students from Level 200 to Level 400, enroled in procurement-related degree programmes at selected public universities, participated in the study. Level 100 students were excluded due to their limited exposure to procurement concepts. The final sample size was deemed adequate for psychometric evaluation, representing an infinite population exceeding 50,000 [51].

In addition, eight procurement experts, comprising academics and industry professionals, were purposively selected to participate in the content validation stage. These experts assessed the initial items for clarity, relevance and representativeness during the instrument development process.

### 6.3. Instrument development and expert review

We started with the development of 40 procurement literacy instruments covering six hypothesised domains informed by the Ghana Public Procurement Act (Act 663, amended by Act 914 and Act 1139), the OECD Recommendation on Public Procurement [21], and the CIPS Global Standard for Procurement and Supply [22]. While these frameworks; the Act and policies informed the development of the instruments, the items were not directly adapted from existing instruments but were newly constructed through expert review to reflect undergraduate learning outcomes. The instruments were grouped into the six domains as follows: Legal and Policy Knowledge (7 items), Procurement Planning and Decision-Making (7 items), Supplier and Contract Management (7 items), Transparency and Accountability (6 items), Ethical Procurement Practices (7 items) and Digital and E-Procurement Competence (6 items). The Item construction was guided by the principle that procurement literacy reflects the ability to interpret, evaluate, and respond to procurement-related situations rather than simply recalling procedural rules. As such, items were framed to capture students’ perceived capability in planning decisions, ethical judgement, supplier oversight, and digital engagement. This approach aligns with competency-based education, which emphasises demonstrable capability, and with constructivist views of learning that prioritise meaning-making through experience. Expert reviewers were specifically asked to assess whether the items reflected realistic procurement contexts and decision processes expected at the undergraduate level.

The content validity of the initial items was assessed using eight academic and industry procurement experts using a four-point scale (1 = not relevant, 2 = somewhat relevant, 3 = relevant,4 = very relevant). The Item-Level Content Validity Index (I-CVI) and Scale-Level CVI (S-CVI/Ave) were calculated [52], and items scoring below 0.78 were revised or excluded [53], and the resultant items were piloted, leading to minor linguistic adjustments to improve clarity and relevance [54].

### 6.4. Data collection procedure

The final instrument was administered through Google Forms to Levels 200, 300 and 400 of selected public universities in Ghana from 18September 2025 to 25 September 2025. The instrument included a preamble explaining the purpose of the study, confidentiality assurance, and voluntary participation. Measures were taken to ensure data quality, including checking for duplicates and completion thresholds.

### 6.5. Psychometric calidation procedures

#### 6.5.1. Exploratory factor analysis (EFA).

The study employed Exploratory factor Analysis (EFA) to examine the underlying structure of the instrument. The EFA utilised the Principal Axis Factoring with Promax rotation, based on the assumption of correlated factors [55](Costello & Osborne, 2005), and three considerations were considered to retain a factor: eigenvalues ≥ 1, scree plot inspection, and theoretical interpretability. All items with factor loadings < 0.50 or high cross-loadings were excluded [56], and the final retained factor were subjected to the confirmatory factor analysis.

#### 6.5.2. Confirmatory factor analysis (CFA).

To cross-validate the emergent structure from the EFA, we used Confirmatory Factor Analysis (CFA) using the lavaan package in R. We evaluated the model using the Comparative Fit Index (CFI), Tucker–Lewis Index (TLI), Root Mean Square Error of Approximation (RMSEA) and Standardised Root Mean Square Residual (SRMR). In line with the threshold recommended by [57,58], *CFI ≥ 0.9, TLI ≥ 0.9, RMSEA ≥ 0.08 and SRMR ≥ 0.08* respectively indicate a reasonable fit.

#### 6.5.3. Convergent validity and construct reliability.

The convergent validity and construct reliability for each factor were assessed using the Average Variance Extracted (AVE) and Composite Reliability (CR). Following Fornell and Larcker [59] (1981) and [60], AVE ≥ 0.50 and CR ≥ 0.70 were used to establish convergent validity and internal construct reliability.

#### 6.5.4. Measurement invariance.

To ensure that the scale functioned equivalently across academic levels (Level 200, 300, 400), a multi-group CFA was conducted to assess configural, metric and scalar invariance [61,62]. Invariance supported the appropriateness of comparing procurement literacy scores across different year groups. This was supported with one-way ANOVA tests conducted for each of the procurement literacy constructs to further evaluate the instrument’s ability to differentiate between academic levels.

### 6.6. Common method bias

To address concerns related to common method bias, both procedural and statistical remedies were implemented. Procedurally, participants were assured of anonymity and confidentiality to reduce evaluation apprehension and social desirability bias. Items were also designed to minimise ambiguity and common rater effects [63]. (Podsakoff et al., 2003).

Statistically, the Harman’s single-factor test was performed to determine whether a single latent factor accounted for the majority of the variance. The analysis revealed that the first unrotated factor explained 48.5% of the total variance, which is below the conventional threshold of 50%, indicating that common method bias was not a significant threat to the integrity of the data [63,64].

### 6.7. Ethical considerations

Ethical approval was obtained from the Committee on Human Research, Publications and Ethics (CHRPE), School of Medical Sciences, Kwame Nkrumah University of Science and Technology, and Komfo Anokye Teaching Hospital, Kumasi, Ghana (Ref: CHRPE/AP/1000/25). Informed consent was obtained from all participants prior to their participation in the study. Consent was documented electronically via a mandatory consent page within the Google Form survey

## 7. Results and discussion

### 7.1. Content validity evaluation

We started the content validation by allowing a panel of eight experts in procurement and education to evaluate the initial 40-item instrument using a 4-point scale ranging from 1 (not relevant) to 4 (highly relevant). Following [52] guidelines, items with an Item-Level Content Validity Index (I-CVI) of 0.78 or higher were retained. Table 2 shows the results of I-CVI and S-CVI/UA of the 40 items. Based on the results, item LEG7, a component of Policy and Legal Knowledge, was deleted, bringing the items to 39. The Scale-Level Content Validity Index based on Universal Agreement (S-CVI/UA) was calculated at 0.925, surpassing the recommended threshold of 0.80 for newly developed instruments [65].

**Table 2:** Content Validity Ratings by Expert Panel (I-CVI and S-CVI).

Item Code	Item	I-CVI
	**Section A: Policy and Legal Knowledge (LEG)**	
LEG1	I can identify the key principles of the public procurement law in my country.	100%
LEG2	I understand the differences between open tendering and restricted tendering.	100%
LEG3	I am familiar with the thresholds that determine procurement methods.	100%
LEG4	I can explain the functions of key entities involved in procurement regulation.	100%
LEG5	I am aware of the role of procurement in promoting national development goals.	88%
LEG6	I understand how international procurement laws (e.g., WTO, UNCITRAL) relate to local laws.	100%
LEG7	I know the penalties associated with procurement fraud or non-compliance.	70%
	**Section B: Procurement Planning and Decision-Making (PLAN)**	
PLAN1	I can develop a procurement plan based on organisational needs.	100%
PLAN2	I can estimate procurement timelines and delivery schedules accurately.	100%
PLAN3	I am confident in selecting appropriate procurement methods for different purchases.	100%
PLAN4	I know how to conduct a needs assessment for procurement.	100%
PLAN5	I can determine procurement specifications that meet value-for-money principles.	100%
PLAN6	I understand how to manage risks in procurement planning.	100%
PLAN7	I know how to forecast budget requirements for procurement activities.	100%
	**Section C: Supplier and Contract Management (SUP)**	
SUP1	I can evaluate supplier bids using pre-defined criteria.	100%
SUP2	I understand how to draft and interpret key clauses in procurement contracts.	100%
SUP3	I know how to manage supplier relationships throughout the contract lifecycle.	100%
SUP4	I am confident in applying dispute resolution mechanisms in procurement contracts.	100%
SUP5	I can monitor supplier performance using key performance indicators (KPIs).	100%
SUP6	I understand contract termination conditions and procedures.	100%
SUP7	I am familiar with contract variation and amendment processes.	100%
	**Section D: Transparency and Accountability (TRAN)**	
TRAN1	I understand the importance of documentation in procurement transparency.	100%
TRAN2	I know how to conduct a procurement process that ensures fairness and equity.	100%
TRAN3	I am familiar with whistleblower mechanisms in public procurement.	88%
TRAN4	I can identify red flags that may indicate procurement malpractice.	100%
TRAN5	I understand the role of audits and performance reviews in procurement accountability.	100%
TRAN6	I believe procurement decisions should be justified and recorded for accountability.	100%
	**Section E: Ethical Procurement Practice**	
ETH1	I can recognise situations that present ethical dilemmas in procurement.	100%
ETH2	I believe accepting gifts from suppliers can compromise procurement integrity.	100%
ETH3	I would report any unethical behaviour I observe during procurement.	100%
ETH4	I understand the concept of conflict of interest in procurement.	100%
ETH5	I believe ethical procurement protects public trust.	100%
ETH6	I can apply procurement ethics even when under pressure.	100%
ETH7	I feel confident making decisions aligned with ethical procurement codes.	100%
	**Section F: Digital and E-Procurement Competence**	
DIG1	I know how to use e-procurement platforms to publish tender notices.	100%
DIG2	I can evaluate e-submissions from suppliers in digital tendering platforms.	100%
DIG3	I understand the benefits of e-procurement for transparency and efficiency.	100%
DIG4	I can participate in online vendor registration and prequalification processes.	100%
DIG5	I am aware of cybersecurity and data privacy concerns in e-procurement systems.	100%
DIG6	I am confident in managing procurement records in digital formats.	100%
	**Scale-Level Content Validity Index based on Universal Agreement (S-CVI/UA)**	92.5%

The remaining 39 items, along with demographic questions and a measure of ethical intention, were administered to a sample of 554 undergraduate procurement students from Level 200 to Level 400 for initial dimensionality assessment. The responses were recorded on a five-point Likert scale, ranging from 1 = “Not true at all” to 5 = “Very true,” reflecting the extent to which each statement applied to the respondent. Level 100 students were excluded due to minimal course exposure. Table 3 presents the demographic characteristics of the sample. A total of 554 undergraduate procurement students participated in the study. The sample was fairly balanced in terms of gender, with 51.9% identifying as male and 48.1% as female.

**Table 3:** Sample Demographic Characteristics (N = 554).

Demographic Variable	Category	n	%
**Level of Study (LoS)**	Level 200	194	35.0%
	Level 300	214	38.6%
	Level 400	146	26.4%
	*Missing*	0	0.0%
**GPA Range**	Below 2.0	49	9.6%
	2.0–2.4	91	17.7%
	2.5–2.9	145	28.3%
	3.0–3.5	163	31.8%
	3.6–4.0	33	12.7%
	*Missing*	41	7.4%
**Gender**	Female	264	48.1%
	Male	285	51.9%
	*Missing*	5	0.9%
**Work Experience**	Full-time Job	10	1.8%
	Internship	258	47.3%
	No Experience	230	42.1%
	Part-time Job	48	8.8%
	*Missing*	8	1.4%

In terms of academic level, the highest proportion of participants was in Level 300 (38.6%), followed by Level 200 (35.0%) and Level 400 (26.4%).

Regarding academic performance, a wide range of Grade Point Averages (GPA) was reported. Approximately 17.7% of students fell within the 2.0 to 2.4 GPA range, 28.3% with the range of 2.5 to 2.9, while 31.8% reported GPAs between 3.0 and 3.5. Smaller proportions of students reported GPAs below 2.0 (9.6%) and 12.7% above 3.5.

Work experience in procurement-related roles also varied in the sample. Nearly half of the students (47.3%) had undertaken internships, while 42.1% reported no prior work experience. A smaller percentage reported having part-time jobs (8.8%) or full-time work experience (1.8%) in procurement-related areas.

These demographic characteristics indicate that the sample comprises students with diverse academic backgrounds, varying levels of academic achievement, and a range of work experiences. This diversity provides a suitable basis for assessing procurement literacy between different groups.

### 7.2. Descriptive statistics of procurement literacy items

Descriptive statistics were computed for individual items under each procurement literacy construct to examine response patterns and assess data suitability for further analyses. As presented in Table 4, the mean scores across items ranged from 2.54 to 3.67, indicating generally moderate levels of agreement among students regarding procurement competencies.

**Table 4:** Descriptive Statistics of Procurement Literacy Items.

Construct	Item Range	Mean Range	SD Range	Skewness Range	Kurtosis Range
Legal and Policy Knowledge (LEG)	LEG1–LEG6	2.69–3.43	1.15–1.31	−0.36 to +0.24	−0.99 to −0.68
Procurement Planning (PLAN)	PLAN1–PLAN7	3.14–3.34	1.16–1.23	−0.31 to −0.08	−0.86 to −0.74
Supplier & Contract Management (SUP)	SUP1–SUP7	2.96–3.32	1.17–1.26	−0.29 to −0.04	−0.89 to −0.73
Transparency & Accountability (TRAN)	TRAN1–TRAN6	2.54–3.67	0.92–1.25	−0.59 to −0.16	−0.87 to −0.57
Ethical Procurement Practice (ETH)	ETH1–ETH7	3.08–3.59	0.93–1.37	−0.79 to −0.14	−0.98 to −0.23
Digital & E-Procurement (DIG)	DIG1–DIG6	2.89–3.38	1.17–1.26	−0.30 to +0.04	−0.98 to −0.75

Note: All items demonstrated acceptable skewness (within ±1) and kurtosis (within ±2), indicating approximate normality (Kline, 2023).

Among the item-level means, those belonging to the Transparency and Accountability and Ethical Procurement Practice domains were among the highest (item means up to 3.67 and 3.59, respectively), suggesting students expressed relatively strong awareness or endorsement of ethical norms and transparency expectations in procurement. This pattern is not surprising, as ethics and transparency have become central themes in procurement reforms and discourse in Ghana’s public sector [16,66,67].

In contrast, items under the Legal and Policy Knowledge construct recorded lower mean values (minimum item mean = 2.69), possibly reflecting students’ limited exposure to formal legal frameworks or their complexity [16,68].

Standard deviations between items ranged from 0.92 to 1.37, reflecting acceptable levels of variability in likert item responses. Skewness values ranged from −0.79 to +0.24 and kurtosis from −0.99 to −0.23, all within the acceptable range for normality assumptions [58,59]. These values support the use of parametric techniques such as Confirmatory Factor Analysis (CFA) and ANOVA.

### 7.3. Exploratory factor analysis (EFA)

To examine the underlying factor structure of the instrument, an Exploratory Factor Analysis (EFA) was conducted. The Kaiser Meyer Olkin (KMO) measure of sampling adequacy was excellent at 0.954, indicating the data were suitable for factor analysis. Bartlett’s test of sphericity was highly significant (χ²(741) = 19,644.17, p < .001), further confirming the appropriateness of the analysis.

The EFA was performed using Principal Axis Factoring with Promax (oblique) rotation to allow for potential correlations among factors. The analysis yielded a five-factor solution that collectively explained 66.00 per cent of the total variance in the data.

Several of the items of one of the conceptual domain, Transparency and Accountability, exhibited substantial cross-loadings, particularly overlapping with dimensions related to ethics and supplier and contract management. This overlap suggested a lack of conceptual distinctiveness for this factor in the current sample. Given the aim of developing a parsimonious and interpretable measurement model, the Transparency and Accountability domain was removed at this stage. The remaining items formed a coherent five-factor structure that was retained for subsequent confirmatory analysis.Additionally, four items (SUP3, TRAN1, TRAN3, and ETH1) did not load significantly on any of the five retained factors, suggesting poor alignment with the latent constructs. Subsequently, These items were removed, resulting in a refined instrument consisting of 35 items across five well-defined factors. The factors and resulting factor loadings are presented in Table 5.

**Table 5:** Factor Loadings from EFA.

	Factor 1	Factor 2	Factor 3	Factor 4	Factor 5	Uniqueness
ETH5	0.940					0.278
ETH3	0.864					0.253
ETH4	0.814					0.248
ETH6	0.773					0.298
ETH7	0.744					0.308
TRAN6	0.707					0.283
ETH2	0.693					0.421
PLAN4		0.826				0.294
PLAN6		0.796				0.318
PLAN3		0.793				0.249
PLAN2		0.781				0.299
PLAN1		0.741				0.385
PLAN7		0.718				0.330
PLAN5		0.598				0.320
SUP1		0.552				0.343
TRAN3			0.692			0.470
SUP7			0.636			0.370
SUP5			0.611			0.261
SUP6			0.603			0.359
SUP2			0.562			0.306
TRAN5			0.549			0.350
TRAN4			0.539			0.357
SUP4			0.533			0.314
DIG2				0.834		0.236
DIG4				0.813		0.263
DIG6				0.747		0.350
DIG5				0.728		0.386
DIG3				0.615		0.416
DIG1				0.601		0.343
LEG3					0.794	0.350
LEG4					0.774	0.341
LEG5					0.683	0.437
LEG1					0.640	0.538
LEG2					0.632	0.447
LEG6					0.614	0.459
SUP3						0.318
TRAN1						0.321
TRAN2						0.302
ETH1						0.351

*Note.* Applied rotation method is promax.

### 7.4. Reliability and internal consistency

The internal consistency reliability was evaluated using Cronbach’s alpha coefficients. All constructs exhibited high reliability, with alpha values ranging from 0.875 to 0.930. Table 6 shows that Ethical Procurement Practice (α = 0.921), Procurement Planning and Decision-Making (α = 0.930), Supplier and Contract Management (α = 0.913), and Digital and E-Procurement Competency (α = 0.916) demonstrated excellent reliability, while Legal and Policy Knowledge also showed strong reliability (α = 0.875). The overall scale showed excellent internal consistency (α = 0.963), indicating robust measurement consistency across the instrument.

**Table 6:** Cronbach’s Alpha Coefficients per Factor.

	Cronbach α
Ethical Procurement Practice	0.921
Procurement Planning and Decision-Making	0.930
Supplier and Contract Management (SUP)	0.913
Digital and E-Procurement Competence	0.916
Legal and Policy Knowledge	0.875
**Total**	**0.963**

### 7.5. Confirmatory factor analysis (CFA)

Confirmatory Factor Analysis (CFA) was performed using JASP 0.18.3, employing the Maximum Likelihood estimation with robust standard errors to assess the structural validity of the instrument. The initial CFA model, comprising all 39 items, yielded a suboptimal fit (CFI = 0.949, TLI = 0.945, SRMR = 0.056, and RMSEA = 0.085), suggesting the need for model refinement.

Following recommendations of [60,69], the items were iteratively reviewed and removed based on a combination of high modification indices, high residual covariances and theoretical redundancy. Items with overlapping content, poor contribution to the factor, or problematic error covariances were systematically eliminated, with a theoretical justification guiding each decision.

The refined model retained 30 items across five factors, demonstrating a substantial improvement in model fit. The final CFA model met the widely accepted fit criteria proposed by [57]: CFI = 0.959, TLI = 0.955, SRMR = 0.051, and RMSEA = 0.080. These results presented in Table 7 confirm the structural validity of the instrument and support its five-factor solution.

**Table 7:** CFA Model Fit Indices – Initial and Final Models.

	Initial	Final
CFI	0.949	0.959
TLI	0.945	0.955
SRMR	0.056	0.051
RMSEA	0.085	0.080

The standardised factor loadings for the final confirmatory factor analysis (CFA) model are presented in Table 8. All items significantly loaded (p < .001) in their respective constructs, with standardised loadings ranging from 0.719 to 0.924, exceeding the commonly accepted threshold of 0.50 [60]. These results confirm strong convergent validity in all five constructs.

**Table 8:** Final Factor Loadings from CFA.

Factor	Indicator	Std. estimate	Std. Error	z-value	p
Ethical Procurement Practice	ETH2	0.816	0.018	44.438	< .001
	ETH3	0.899	0.011	81.675	< .001
	ETH4	0.924	0.010	95.770	< .001
	ETH5	0.856	0.015	58.337	< .001
	ETH6	0.891	0.012	74.523	< .001
	ETH7	0.911	0.012	78.141	< .001
Procurement Planning and Decision-Making	PLAN1	0.797	0.017	46.465	< .001
	PLAN2	0.894	0.011	80.563	< .001
	PLAN3	0.905	0.010	92.419	< .001
	PLAN4	0.869	0.013	68.114	< .001
	PLAN5	0.883	0.012	71.723	< .001
	PLAN6	0.836	0.015	57.341	< .001
Supplier and Contract Management	SUP1	0.858	0.013	63.917	< .001
	SUP2	0.856	0.013	63.636	< .001
	SUP4	0.867	0.012	72.086	< .001
	SUP5	0.889	0.011	83.211	< .001
	SUP6	0.847	0.014	61.653	< .001
	SUP7	0.817	0.016	52.533	< .001
Digital and E-Procurement Competence	DIG1	0.915	0.014	64.039	< .001
	DIG2	0.865	0.012	70.992	< .001
	DIG3	0.840	0.016	52.598	< .001
	DIG4	0.872	0.012	71.312	< .001
	DIG5	0.815	0.016	50.718	< .001
	DIG6	0.832	0.016	51.384	< .001
Legal and Policy Knowledge	LEG1	0.719	0.028	25.657	< .001
	LEG2	0.747	0.025	30.144	< .001
	LEG3	0.802	0.020	39.431	< .001
	LEG4	0.845	0.018	47.972	< .001
	LEG5	0.801	0.021	38.378	< .001
	LEG6	0.783	0.025	31.888	< .001

**Fig 1:**
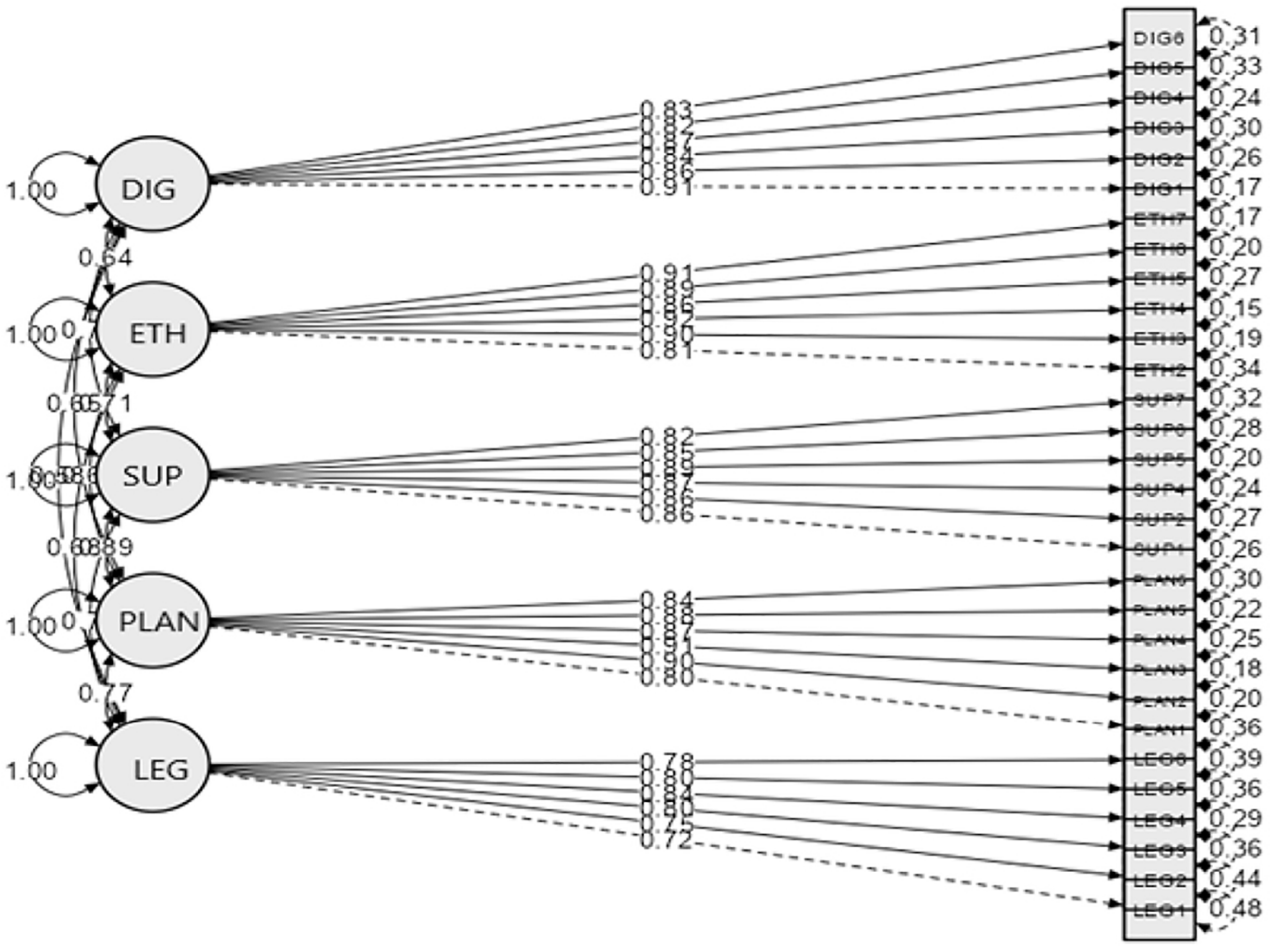
CFA Path Diagram (Final 5-Factor, 30-Item Model).

Fig 1 presents the final Confirmatory Factor Analysis (CFA) path diagram for the 30-item, five-factor model. The diagram displays the standardised regression weights, residual variances, and inter-factor covariances among the latent constructs: Ethical Procurement Practice (ETH), Procurement Planning and Decision-Making (PLAN), Supplier and Contract Management (SUP), Digital and E-Procurement Competence (DIG), and Legal and Policy Knowledge (LEG). The factor loadings in the diagram correspond to the values reported in Table 8, visually confirming the strength and clarity of the measurement model [60,69].

All latent constructs demonstrated strong associations with their respective items, with low residual variances and no substantial cross-loadings, thereby supporting the internal consistency of the model. The inter-factor correlations ranged from 0.64 to 0.89. This indicates that while the constructs are conceptually related, which is expected within the multidimensional framework of procurement literacy, they remain sufficiently distinct to be treated as separate dimensions. This pattern supports discriminant validity in line with the criteria proposed by [59] and later extended by [70].

The final CFA model therefore validates the proposed five-factor structure of procurement literacy and offers strong empirical evidence for the psychometric robustness of the instrument. Clear factor loadings and meaningful correlations among the constructs provide a solid foundation for the application of the instrument in further analyses of procurement knowledge and competency between student populations [58,71].

The alignment between the theoretical framework and the final empirical structure of the instrument strengthens confidence in its validity. The retained dimensions reflect capability areas that are both pedagogically meaningful and practically relevant, supporting the view that procurement literacy at the undergraduate level is best understood as a blend of applied knowledge, ethical orientation, and functional readiness. The coherence between theory, item design, and factor structure suggests that the instrument captures how procurement competence is cognitively organised during early professional formation.

### 7.6. Convergent and discriminant validity

Convergent validity was evaluated by calculating the Average Variance Extracted (AVE) for each construct. All constructs exceeded the recommended threshold of 0.50 [59], confirming adequate convergent validity. Table 9 presents AVE values ranging from 0.614 to 0.781, with particularly strong results observed for Ethical Procurement Practice (AVE = 0.781), Procurement Planning and Decision-Making (AVE = 0.748), and Supplier and Contract Management (AVE = 0.723). Legal and Policy Knowledge also demonstrated satisfactory convergent validity (AVE = 0.614).

**Table 9:** Average variance extracted.

Factor	AVE
Ethical Procurement Practice	0.781
Procurement Planning and Decision-Making	0.748
Supplier and Contract Management (SUP)	0.723
Digital and E-Procurement Competence	0.735
Legal and Policy Knowledge	0.614

Discriminant validity was assessed using the Heterotrait-Monotrait (HTMT) ratio of correlations and presented in Table 10. All HTMT values fell below the conservative threshold of 0.90 [70], indicating adequate discriminant validity across constructs. The highest HTMT value was found between Procurement Planning and Decision-Making and Supplier and Contract Management (HTMT = 0.888), which, although within acceptable limits, suggests some conceptual overlap that warrants careful interpretation.

**Table 10:** Heterotrait-monotrait ratio.

Ethical Procurement Practice	Procurement Planning and Decision-Making	Supplier and Contract Management (SUP)	Digital and E-Procurement Competence	Legal and Policy Knowledge
1.000				
0.656	1.000			
0.709	0.888	1.000		
0.589	0.639	0.711	1.000	
0.657	0.762	0.752	0.583	1.000

To justify group comparisons, measurement invariance across academic levels (Levels 200, 300, and 400) was assessed using multi-group Confirmatory Factor Analysis (CFA). The results supported configural, metric, and scalar invariance, indicating that the factor structure, item loadings, and intercepts were equivalent across groups [61], which confirms that procurement literacy scores are meaningfully comparable across academic levels.

To further evaluate the instrument’s ability to differentiate between academic levels, one-way ANOVA tests were conducted for each of the five procurement literacy constructs. The analysis presented in Table 11 revealed significant differences between academic levels for all constructs.

**Table 11:** Results of One-Way ANOVA for five dimensions of Procurement Literacy against Academic Levels.

Construct	F(df)	p-value	η² (Effect Size)	Conclusion
Ethical Procurement Practice	F(2, 519)= 9.26	<.01	0.034	Significant group differences
Procurement Planning and Decision-Making	F(2, 541) = 5.89	<.01	0.021	Significant group differences
Supplier and Contract Management	F(2, 529) = 8.12	<.01	0.028	Significant group differences
Digital and E-Procurement Competence	F(2, 531) = 6.21	<.01	0.022	Significant group differences
Legal and Policy Knowledge	F(2, 530)= 14.15	<.01	0.051	Significant group differences

Specifically, significant group differences were observed for Ethical Procurement Practice (F(2, 519)= 9.26, p < .01, η² = 0.034), Procurement Planning and Decision-Making (F(2, 541) = 5.89, p < .01, η² = 0.021), Supplier and Contract Management (F(2, 529) = 8.12, p < .01, η² = 0.022), Digital and E-Procurement Competence (F(2, 531) = 6.21, p < .01, η² = 0.028), and Legal and Policy Knowledge (F(2, 530)= 14.15, p < .01, η² = 0.051). These findings indicate that procurement literacy varies significantly across academic levels for all dimensions measured by the instrument.

The effect sizes (η²) ranged from 0.021 to 0.051, representing small to moderate effects according to conventional benchmarks [72,73]. These results suggest that students’ procurement literacy generally increases with higher academic exposure and experience across all competency areas.

### 7.7. Predictive validity

To evaluate predictive validity, Pearson correlation analyses were conducted between the procurement literacy constructs and the students’ behavioural intention to engage in ethical procurement practices. The results presented in Table 12 showed that all correlations were statistically significant (p < .001), which support for the predictive validity of the instrument.

**Table 12:** Correlations between Dimension of Procurement Literacy and Ethical Behavioural Intention.

	Pairings		Pearson’s r		p	Effect size (Fisher’s z)	SE Effect size
PIE	–	PLAN	0.616	***	< .001	0.719	0.044
PIE	–	SUP	0.638	***	< .001	0.755	0.045
PIE	–	LEG	0.607	***	< .001	0.704	0.045
PIE	–	DIG	0.552	***	< .001	0.622	0.044
PIE	–	BEH	0.823	***	< .001	1.165	0.045
PLAN	–	SUP	0.829	***	< .001	1.185	0.044
PLAN	–	LEG	0.695	***	< .001	0.858	0.044
PLAN	–	DIG	0.590	***	< .001	0.678	0.044
PLAN	–	BEH	0.589	***	< .001	0.676	0.044
SUP	–	LEG	0.669	***	< .001	0.809	0.044
SUP	–	DIG	0.616	***	< .001	0.718	0.044
SUP	–	BEH	0.576	***	< .001	0.657	0.044
LEG	–	DIG	0.512	***	< .001	0.565	0.044
LEG	–	BEH	0.568	***	< .001	0.645	0.045
DIG	–	BEH	0.462	***	< .001	0.500	0.044

* p < .05, ** p < .01, *** p < .001.

In particular, the behavioural intention of the students demonstrated strong positive associations with all five procurement literacy dimensions. Among these, Supplier and Contract Management showed a robust correlation with behavioural intention (r = 0.638), followed closely by Procurement Planning and Decision-Making (r = 0.616), Legal and Policy Knowledge (r = 0.607), and Digital and E-Procurement Competence (r = 0.552). The strongest relationship was observed between behavioural intention and Ethical Procurement Practice (r = 0.823), suggesting that students’ ethical dispositions are highly predictive of their intention to engage in ethical procurement actions.

Beyond these associations, the dimensions of procurement literacy themselves were also strongly interrelated, with correlation coefficients ranging from 0.462 to 0.829. This pattern reinforces the conceptual coherence of the constructs and highlights their collective contribution to predicting ethical procurement behaviours. Together, these findings provide compelling evidence for predictive validity ofthe instrument.

### 7.8. Discussions

This study provides robust empirical support for a five-factor, 30-item instrument designed to measure procurement literacy among undergraduate students. The development and validation process adhered to established psychometric principles, combining exploratory and confirmatory factor analyses to ensure structural validity, convergent and discriminant validity, and internal consistency. The iterative refinement of the instrument, although requiring the removal of several items, was guided by best practices in scale development, prioritising theoretical alignment and model parsimony over item retention [74,75]. While some items demonstrated acceptable loadings during exploratory factor analysis, they were subsequently excluded based on their adverse impact on the global model fit during confirmatory factor analysis. This trade-off is consistent with established recommendations that underscore the importance of model fit and conceptual clarity in instrument refinement [69].

The resulting instrument offers a multidimensional conceptualisation of procurement literacy, comprising Ethical Procurement Practice, Procurement Planning and Decision Making, Supplier and Contract Management, Digital and Electronic Procurement Competence, and Legal and Policy Knowledge. These dimensions not only reflect core procurement competencies, but also align with Levels 2–4 of the Chartered Institute of Procurement and Supply (CIPS) Global Standard [22] enhancing the tool’s relevance for academic benchmarking and professional alignment.

Importantly, the inclusion of ethical disposition and behavioural intention constructs in the validation process adds conceptual depth to the instrument, acknowledging the normative dimensions of procurement decision making. As supported by the literature, ethical reasoning and commitment are central to professional integrity and accountability in procurement environments [43,76]. Strong predictive relationships observed between the procurement literacy dimensions and students’ intention to engage in ethical procurement practices underscore the applied value of the instrument in assessing not only knowledge but also ethical orientation.

From a pedagogical standpoint, the validated instrument holds substantial potential for use in curriculum design, diagnostics, and monitoring of learning outcomes. Its ability to capture core and evolving competencies such as digital proficiency and regulatory awareness positions it as a relevant framework for educational institutions seeking to strengthen procurement training. Additionally, the ability of the instrument to detect group differences across academic levels further reinforces its diagnostic utility, allowing educators to track student development over time and design targeted interventions where gaps in competencies are identified.

In summary, the final validated procurement literacy instrument is theoretically grounded, psychometrically sound, and practically useful. It offers a valuable resource for improving procurement education in higher education settings and can serve as a foundational tool for future studies examining procurement competence, ethical practices, and behavioural intentions in academic or professional contexts.

## 8. Conclusion

This study developed and validated a psychometrically robust instrument for assessing procurement literacy among undergraduate students. Grounded in contemporary procurement theory and aligned with professional competency frameworks, the instrument captures five core dimensions: Ethical Procurement Practice, Procurement Planning and Decision Making, Supplier and Contract Management, Digital and Electronic Procurement Competence, and Legal and Policy Knowledge. Through rigorous exploratory and confirmatory factor analyses, the final 30-item scale (see S1 Appendix) demonstrated excellent structural validity, strong internal consistency, and sound convergent and discriminant properties.

Beyond measurement reliability, the instrument exhibits predictive validity by meaningfully correlating with behavioural intentions of students to engage in ethical procurement practices. This connection underscores the practical importance of procurement literacy not only as a technical competency but also as a normative framework for fostering integrity and accountability in public and private sector procurement. The instrument’s ability to differentiate student performance across academic levels further enhances its relevance for pedagogy, curriculum evaluation, and tracking of student progression.

The findings have several implications for procurement education. First, the validated instrument offers a foundation for benchmarking and standard setting in procurement training at the tertiary level. Second, it provides educators and curriculum developers with a reliable tool for diagnosing competency gaps and aligning instruction with both academic and industry needs. Lastly, by integrating ethical and digital competencies, the instrument supports the broader goal of preparing procurement professionals who are both technically competent and ethically grounded.

Future research may extend this work by testing the instrument across diverse institutional and cultural contexts, exploring longitudinal changes in student competence, and examining the influence of targeted interventions on procurement learning outcomes. Such studies will further consolidate the instrument’s utility and contribute to the global discourse on procurement capacity building in higher education.

## Supporting information

S1 Apppendix Final validated questionnaire. This appendix contains the final validated procurement literacy questionnaire used in the study.
(DOCX)
