# Supplementary material for: A multidimensional procurement literacy instrument: Development and validation among undergraduate procurement students in Ghana
Source: PLoS One. 2026 Feb 3;21(2):e0341565. doi: 10.1371/journal.pone.0341565 (PMC12867244; doi:10.1371/journal.pone.0341565)
Supplement: S1 Apppendix — This appendix contains the final validated procurement literacy questionnaire used in the study. (DOCX) [file pone.0341565.s001.docx]

**S1 Appendix**

**Final Validated Multidimmension Procurement Literacy Instrunents**

| S/N | Dimensions and Items |
| --- | --- |
| **Policy and Legal Framework Knowledge** | |
|  | I can identify the key principles of the public procurement law in my country. |
|  | I understand the differences between open tendering and restricted tendering. |
|  | I am familiar with the thresholds that determine procurement methods. |
|  | I can explain the functions of key entities involved in procurement regulation. |
|  | I am aware of the role of procurement in promoting national development goals. |
|  | I understand how international procurement laws (e.g., WTO, UNCITRAL) relate to local laws. |
| **Procurement Planning and Decision-Making** | |
|  | I can develop a procurement plan based on organisational needs. |
|  | I can estimate procurement timelines and delivery schedules accurately. |
|  | I am confident in selecting appropriate procurement methods for different purchases. |
|  | I know how to conduct a needs assessment for procurement. |
|  | I can determine procurement specifications that meet value-for-money principles. |
|  | I understand how to manage risks in procurement planning. |
| **Supplier and Contract Management** | |
|  | I can evaluate supplier bids using pre-defined criteria. |
|  | I understand how to draft and interpret key clauses in procurement contracts. |
|  | I am confident in applying dispute resolution mechanisms in procurement contracts. |
|  | I can monitor supplier performance using key performance indicators (KPIs). |
|  | I understand contract termination conditions and procedures. |
|  | I am familiar with contract variation and amendment processes. |
| **Ethical Procurement Practice** | |
|  | I believe accepting gifts from suppliers can compromise procurement integrity. |
|  | I would report any unethical behaviour I observe during procurement. |
|  | I understand the concept of conflict of interest in procurement. |
|  | I believe ethical procurement protects public trust. |
|  | I can apply procurement ethics even when under pressure. |
|  | I feel confident making decisions aligned with ethical procurement codes. |
| **Digital and E-Procurement Competence** | |
|  | I know how to use e-procurement platforms to publish tender notices. |
|  | I can evaluate e-submissions from suppliers in digital tendering platforms. |
|  | I understand the benefits of e-procurement for transparency and efficiency. |
|  | I can participate in online vendor registration and prequalification processes. |
|  | I am aware of cybersecurity and data privacy concerns in e-procurement systems. |
|  | I am confident in managing procurement records in digital formats. |
